# SEOM clinical guidelines on cardiovascular toxicity (2018)

**DOI:** 10.1007/s12094-018-02017-3

**Published:** 2019-01-09

**Authors:** J. A. Virizuela, A. M. García, R. de las Peñas, A. Santaballa, R. Andrés, C. Beato, S. de la Cruz, J. Gavilá, S. González-Santiago, T. L. Fernández

**Affiliations:** 1 Servicio de Oncología Médica, Complejo Hospitalario Regional Virgen Macarena, Av. Doctor Fedriani, 3, 41009 Seville, Spain; 2 Servicio de Cardiología, Complejo Asistencial Universitario de Salamanca (CAUSA), Instituto de Investigación Biomédica de Salamanca (IBSAL), Ciber CV, Salamanca, Spain; 3 Servicio de Oncología Médica, Hospital Provincial de Castellón, Castellón de la Plana, Spain; 4 Servicio de Oncología Médica, Hospital Universitari I Politècnic la Fe, Valencia, Spain; 5 Servicio de Oncología Médica, Hospital Clínico Universitario Lozano Blesa, Zaragoza, Spain; 6 Servicio de Oncología Médica, Complejo Hospitalario Regional Virgen Macarena, Sevilla, Spain; 7 Servicio de Oncología Médica, Complejo Hospitalario de Navarra, Pamplona, Spain; 8 Servicio de Oncología Médica, Fundación Instituto Valenciano de Oncología (IVO), Valencia, Spain; 9 Servicio de Oncología Médica, Complejo Hospitalario Universitario de Cáceres, Cáceres, Spain; 10 Servicio de Cardiología, Hospital Universitario La Paz, IdiPAZ, CiberCV, Madrid, Spain

**Keywords:** Chemotherapy, Cardiotoxicity, Early detection, Risk assessment, Cancer

## Abstract

One of the most common side effects of cancer treatment is cardiovascular disease, which substantially impacts long-term survivor’s prognosis. Cardiotoxicity can be related with either a direct side effect of antitumor therapies or an accelerated development of cardiovascular diseases in the presence of preexisting risk factors. Even though it is widely recognized as an alarming clinical problem, scientific evidence is scarce in the management of these complications in cancer patients. Consequently, current recommendations are based on expert consensus. This Guideline represents SEOM’s ongoing commitment to progressing and improving supportive care for cancer patients.

## Introduction

Cardiovascular (CV) diseases compete with second malignancies as the leading cause of mortality in cancer survivors. Antineoplastic treatments nearly triple the risk for CV events over the medium and long term [1]. Clinical management of these toxicities with the aid of multidisciplinary protocols for prevention, diagnosis, and treatment, decreases unnecessary antitumor treatment discontinuation and optimizes global patient’s outcomes [2].

## Cardiotoxicity risk stratification

Although we do not have evidence-based prospective cardiotoxicity scores to stratify the risk for cancer treatment-related CV complications, data from clinical trials and real-life registries enable us to recognize populations at increased risk. Table 1 summarizes the most common variables increasing cardiotoxicity risk [3–5].

**Table 1:**
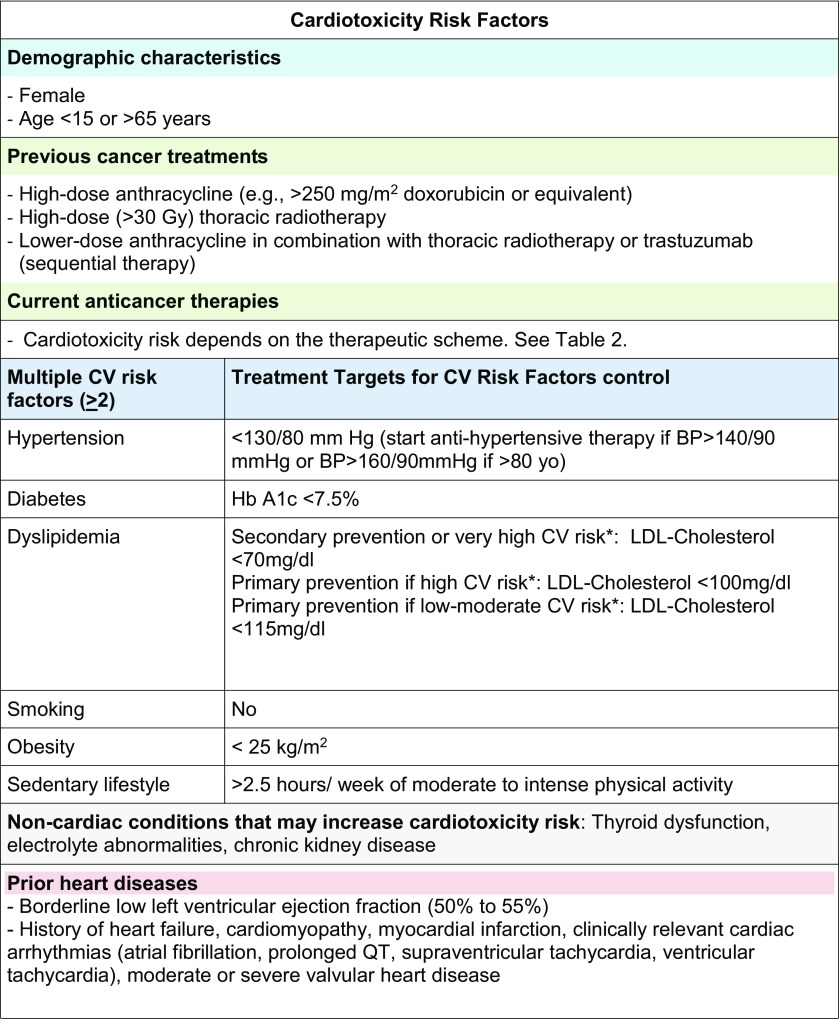
Cardiotoxicity risk factors

*BP* blood pressure, *CV* Cardiovascuar

*https://heartscore.escardio.org/2012/calc.aspx?model=europelow

## Preventive strategies

The prevention of cardiotoxicity begins before cancer therapy, with the cardiologist and the oncologist working together to stratify baseline risk and to decide the best therapeutic approach for each patient. Additionally routine periodic assessment of cardiac function is recommended during and at the end of treatment according to local multidisciplinary protocols [6]. As general rules:

At baseline, minimizing the use of potentially cardiotoxic therapies, if established alternatives would not compromise outcome, is critical to decrease CV events [7, 8]. Optimization of CV risk factors and previous CV conditions is mandatory, as well as a structured advice regarding a healthy lifestyle (diet, smoking and exercise). CV risk stratification with the EAPC’S HeartScore (https://heartscore.escardio.org/2012/calc.aspx?model=europelow) helps to define specific therapeutic goals for CV risk factors’ control (Table 1) [3, 4, 9]. Baseline echocardiography, in patients at risk for heart failure (HF), helps to optimize CV therapy and to define individuals requiring a close supervision during therapy [10]. Universal primary prevention based on standard HF drugs is controversial and only small studies have demonstrated clinical benefit in high-risk populations [11–14].

During and after therapy, continuing CV risk control and active CV monitoring are critical issues. Cardiac biomarkers (e.g., cardiac troponins) and echocardiography in patients at risk for HF allow for early detection and treatment of subclinical myocardial damage, thereby preventing further events [15].

## Monitoring and diagnosis of cardiotoxicity

We define cardiotoxicity as any cancer treatment-related CV event. Diagnostic criteria are similar to those used for general population, with the exception of cardiac dysfunction due to antitumor medication that is defined as a decrease in left ventricular ejection fraction (LVEF) > 10% from baseline to a final LVEF below the lower limit of normal (< 53%) [4, 16]. Table 2 summarizes the most common CV side effects of anticancer therapies [17]. Baseline evaluation before potentially cardiotoxic treatments should include the following:

**Table 2:** Cardiovascular toxicity due to antineoplastic drugs

Cardiovascular toxicity	Associated drugs
Heart failure	Doxorubicin, daunorubicin, idarubicin, epirubicin, mitoxantrone Cyclophosphamide, ifosfamide Docetaxel Trastuzumab, bevacizumab Sunitinib, pazopanib, sorafenib, imatinib, dasatinib, lapatinib, nilotinib Carfilzomib, Bortezomib
Myopericarditis	Cyclophosphamide 5-fluorouracil, cytarabine Trastuzumab, rituximab Interleukin-2 Immune-checkpoint inhibitors
Ischemic cardiomyopathy	5-fluorouracil, capecitabine Cisplatin Paclitaxel, docetaxel Etoposide Bebacizumab Sorafenib, sunitinib Bleomycin
Atrial fibrillation	Cisplatin Cyclophosphamide, ifosfamide, melphalan Doxorubicin Capecitabine, 5-FU Gemcitabine Etoposide Paclitaxel Rituximab Sorafenib, sunitinib, ibrutinib Bortezomib Interleukin-2, interferon
Bradyarrhythmias	Cisplatin Cyclophosphamide, ifosfamide Doxorubicin, epirubicin, mitoxantrone Capecitanina, 5-FU Gemcitabine Paclitaxel Thalidomide Imatinib, bortezomib Rituximab Arsenic trioxide, interleukin-2
Accelerated atherosclerosis	Bevacizumab, nilotinib, ponatinib Carfilzomib, bortezomib
Pericardial effusion	Cyclophosphamide Immune-checkpoint inhibitors^1^
Venous thromboembolic disease	5-fluorouracil Cisplatin Nilotinib, ponatinib, erlotinib Bevacizumab Vorinostat L-Asparaginase Immune-checkpoint inhibitors
Arterial thromboembolic disease	Cisplatin, carboplatin Gemcitabine Bleomycin Vincristine Nilotinib, ponatinib Bevacizumab Interferon alfa-2 Immune-checkpoint inhibitors
Arterial hypertension	Bevacizumab Sorafenib, sunitinib, axitinib, vandetanib, regorafenib
Pulmonary hypertension	Dasatinib Cyclophosphamide
Prolonged QT interval	Doxorubicin Depsipeptide, vorinostat Axitinib, cabozantinib, crizotinib, dasatinib, lapatinib, nilotinib, sorafenib, sunitinib, vandetanib, vemurafenib, ribociclib Arsenic trioxide

**Fig. 1:**
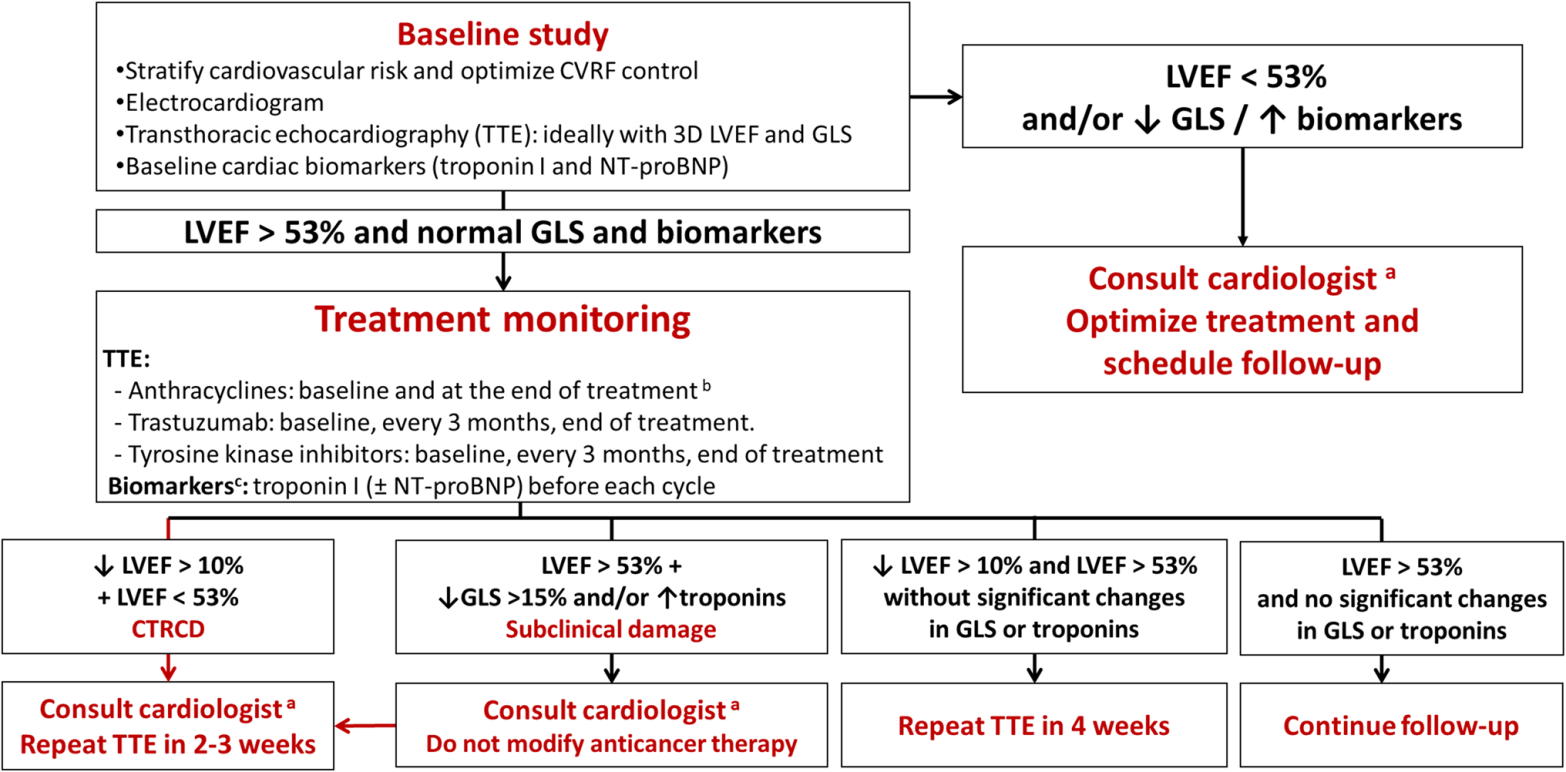
Monitoring algorithm in patients receiving drugs at risk of heart failure. Modified from [4]. *3D* 3-dimensional, *CTRCD* cancer therapeutics-related cardiac dysfunction, *CVRFs* cardiovascular risk factors, *GLS* global longitudinal strain, *LVEF* left ventricular ejection fraction, *NT-proBNP* N-terminal pro-B type natriuretic peptide, *TTE* transthoracic echocardiography. Ideally, a specialist cardio-onco-hematology clinic. **b** Reevaluation of LVEF is recommended before treatment completion if the cumulative dose exceeds 240 mg/m^2^. In these patients, the LVEF should be regularly monitored until the end of treatment. **c** In patients with low cardiovascular risk and without history of cardiotoxic treatment, determination of troponin levels before each cycle reduces the number of echocardiograms required and limits their use to symptomatic patients or those with troponin elevation

- History and physical examination: recording the presence of cardiovascular risk factors (CVRF), preexisitng structural heart disease (Table 1), and prior cardiotoxic treatments [3, 4].
- Electrocardiogram (ECG): if abnormal a cardio-oncologyconsultation is recommended [18].
- Cardiac biomarkers: cardiac troponins are considered an alternative to serial echocardiograms in the ESMO Guidelines for cancer treatment monitoring in patients under anthracyclines ± trastuzumab or tyrosine kinase inhibitors [19–21]. Baseline values are needed to evaluate significant changes during follow-up.
- Imaging techniques: echocardiography is deemed the technique of choice when undertaking a global comprehensive assessment of cardiac structure and function at baseline and during the cancer process. In patients with poor image quality, cardiac magnetic resonance is the best option to avoid the radiation associated with nuclear medicine techniques [3, 4, 16]. Monitoring protocols during treatment should be adapted to both the availability of local resources and the professionals’ expertise [3, 4] to avoid unjustified delays in cancer treatment. Figure 1 summarizes the monitoring process in patients at risk for developing heart failure.

**Table 3:** Final recommendations

Recommendations	Strength of recommendation	Quality of evidence
1. Cardiotoxicity risk stratification		
Patients with cancer who need any potentially cardiotoxic drug should be screened for their cardiotoxicity risk	A	III
Patients with previous cardiovascular disease, prior cardiotoxic treatments, and uncontrolled cardiovascular risk factors should be considered at high risk for cardiotoxicity	A	III
Patients at high risk for cardiotoxicity should be referred for cardiovascular evaluation (ideally cardio-oncology evaluation) before antineoplastic treatment	A	III
2. Preventive strategies for cardiotoxicity in patients at risk		
Minimize the use of potentially cardiotoxic therapies	A	III
All cancer patients should receive recommendations for healthy lifestyle and physical exercise	A	III
Optimize cardiovascular risk factors and previous cardiovascular diseases treatment before, during, and after oncological therapy	A	III
3. Cardiotoxicity diagnosis		
Echocardiography is the imaging technique of choice for the diagnosis and treatment of cancer related cardiovascular complications	A	III
High-risk patients should undergo more intensive follow-up, referring them, for specific Cardio-Oncology consultation	A	III
4. Heart failure monitoring and management		
LV function monitoring should be performed using the same imaging technique during follow-up (2D echo, 3D echo or strain). The choice should be based on center’s availability and clinician’s expertise	A	III
Cardiac biomarkers helps heart failure monitoring	A	II
Patients with a LVEF under normal values (53%) should be referred for cardio-oncology evaluation and treatment	A	I
Cancer treatment interruptions must be based on multidisciplinary team discussion after confirming the presence of symptomatic moderate to severe LV dysfunction	A	III
5. Cardiac arrhythmia monitoring and management		
Patients with cancer at risk for cardiac arrhythmias should undergo close monitoring and ECG screening during the first weeks of therapy	A	III
Anticoagulation in patients with atrial fibrillation should be guided by CHA2Ds2-VASc and HASBLED scores	A	III
6. QT interval monitoring and management		
Assessment of patients treated with potential QT-prolonging drugs should include a baseline electrocardiogram and regular monitoring of the cQT interval (Fridericia´s correction formula)		
If corrected QT interval > 500 ms or increases > 60 ms from baseline, antitumoral drugs must be withdrawn or administrated with hospital monitoring. Any modifiable risk factors (electrolyte abnormalities, use of other QT-prolonging drugs, etc.) must be arranged		
7. Ischemic heart disease monitoring and management		
Optimal CVRF control is critical to minimize ischemic events during and after cancer treatment	A	III
8. Pulmonary hypertension monitoring and management		
These patients require multidisciplinary evaluation to determine the best treatment strategy	D	III
9. Pericardial disease monitoring and management		
These patients require multidisciplinary evaluation to determine the best treatment strategy	D	III
10. Monitoring of long-term survivors		
Cardiovascular screening reduces the incidence of heart failure; however, there is no consensus regarding the optimal screening test or frequency of testing	B	III
During follow-up of long-term cancer survivors, lifestyle modifications to prevent cardiovascular risk factors and instruct patients to report early signs and symtoms	B	III
Patients who need treatment should be referred to Cardio-Oncology	B	III

## Cancer treatment-related CV complications

### Myocardial dysfunction and heart failure

Myocardial dysfunction and heart failure (HF) are nowadays the most common recognized cancer therapy-related CV complications and much of the focus has been in the early detection and prevention of HF.

#### Anthracyclines

Doxorubicin is associated with a 5% incidence of congestive HF with a cumulative lifetime dose of 400 mg/m^2.^ Cardiotoxicity risk increases with higher doses (48% at 700 mg/m^2^) [22]. However, recent studies have demonstrated that there is truly no safe dose of anthracyclines and HF rates can be up to 10% with standard doses in patients > 65 years or with preexisting CV risk factors or cardiac diseases [22].

Acute toxicity is rare (1%) and usually manifests as supraventricular arrhythmias, transient LV dysfunction, or electrocardiographic changes (QT prolongation). For a long time it has been considered that subacute anthracycline damage was irreversible. However, active monitoring allows us for early diagnosis of HF and early treatment, ideally in asymptomatic patients, may change the natural history of anthracycline toxicity. Therefore, modern registries found 98% of cases diagnosed during the first year of treatment in asymptomatic patients [4, 20].

#### Other conventional chemotherapies

Cyclophosphamide cardiotoxicity is relatively rare (generally occurring at higher doses > 140 mg/kg) [23]. Cisplatin and ifosfamide are uncommon causes of HF, usually due to volume overload during treatment infusion. Docetaxel also appears to increase HF risk in patients with preexisting cardiac diseases [3].

In several large-scale trials of adjuvant therapy in breast cancer, the rate of trastuzumab-related cardiac dysfunction ranged from 7 to 34%, with HF class III or IV rates between 0 and 4% [24].The risk is higher in patients with preexisting CV diseases or hypertension and lower in anthracycline-free regimens [3].

Anti-VEGF antibody (bevacizumab) induced LV dysfunction in 2% and TKIs (sunitinib, pazopanib and axitinib) in 3–15% [3].

Practical recommendations for patients with symptoms of HF or significant changes in LVEF [3, 4, 25].

1. Confirm echo data of left ventricular dysfunction (repeat echo at 2–3 weeks).
2. Evaluate symptomatic status and check NT-proBNP.
3. If LVEF is < 53% or other pathologic findings are noticed the patients must be referred to the cardio-oncology unit to consider HF therapy and a multidisciplinary discussion is needed to reevaluate cancer treatment strategy.

### Arterial hypertension

New European Society of Cardiology clinical practice guidelines define arterial hypertension as a blood pressure  > 140/90 mmHg in the office or > 130/80 mmHg during ambulatory measurements [26].

Hypertension is the most common comorbidity in cancer patients [3, 4, 21]. It is present in more than 30% of patients, due to both the high prevalence of hypertension in aged cancer populations and the effect of certain anticancer drugs. Oncological therapies cause hypertension through different mechanisms, although the most frequent are drugs that inhibit angiogenesis and interact with vascular endothelial growth factors such as VEGF inhibitors (e.g., bevacizumab), tyrosine kinase inhibitors (e.g., sunitinib), and sorafenib. It has been reported that VEGF inhibitors induce new hypertension or destabilizing previously controlled hypertension in 11–45% of patients [27].

Blood pressure should be monitored before and during cancer treatment and properly managed, following standard pharmacological and dietary recommendations for the general population [3, 4, 26].

Blood pressure target in patients with uncomplicated hypertension is < 130/80 mmHg. Renin-angiotensin system blockers, betablockers and dihydropyridin calcium channel blockers are considered the drugs of choice, given their protective profile against the onset of HF. In uncontrolled patients double or triple therapy is recommended as well as the addition of antialdosteronic agents. Thiazides should be used with caution because of the risk of hypokalemia and QTc prolongation. The use of negative inotropics drugs (diltiazem and verapamil) is not advised as they block the CYP3A4 isoenzyme, which is involved in the metabolic pathway of some tyrosin kinase inhibiotrs like sorafenib [26].

### Cardiac arrhythmias

There are increasing data that a growing number of anticancer drugs could cause pro-arrhythmic cardiotoxicity [18, 28] (Table 2). Cancer therapy might produce electrophysiological changes, such as QT prolongation, as well as a wide range of cardiac arrhythmias, including bradyarrhythmias and supraventricular and ventricular tachycardias [3, 4]. Arrhythmias might be only slightly perceptible, yet cause severe symptoms or even sudden cardiac death. Oncological diseases themselves predispose to the development of arrhythmias, which may be present at baseline in 16–36% of patients [3, 4, 28]. Management of arrhythmias should be based on cardiac- and cancer-related life expectancy, quality of life, and complication risks [3, 4, 28].

**Fig. 2:**
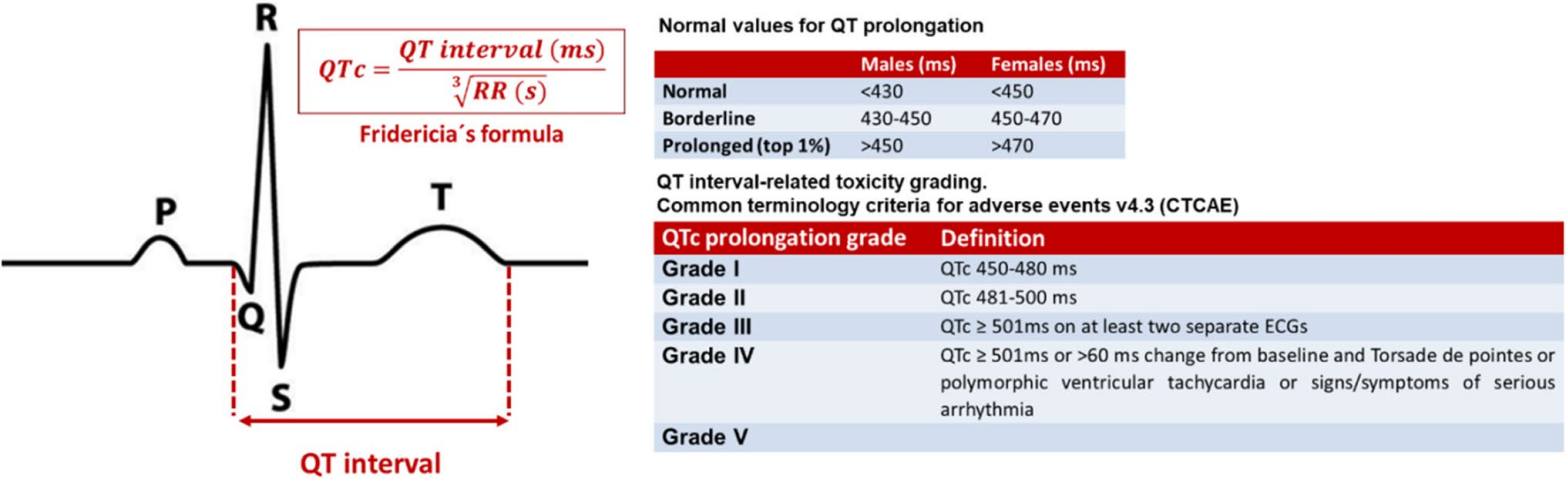
Corrected QT interval calculation using the Fridericia’s formula [18] and QT interval-related toxicity grading. Fridericia’s formula (QTc = QT interval/$\sqrt[3]{RR}$) is the preferred correction formula for oncology population. (*QTc* corrected QT interval, *ms* milliseconds, *s* seconds)

Initial assessment of patients who receive potential QT-prolonging drugs should include a baseline electrocardiogram and regular monitoring of the QT interval during therapy [3, 4, 28, 29] (Fig. 2). Withdrawal of these drugs or administration under hospital monitoring should be considered, if corrected QT interval is > 500 ms or increased by more than 60 ms from baseline [3, 4].

**Fig. 3:**
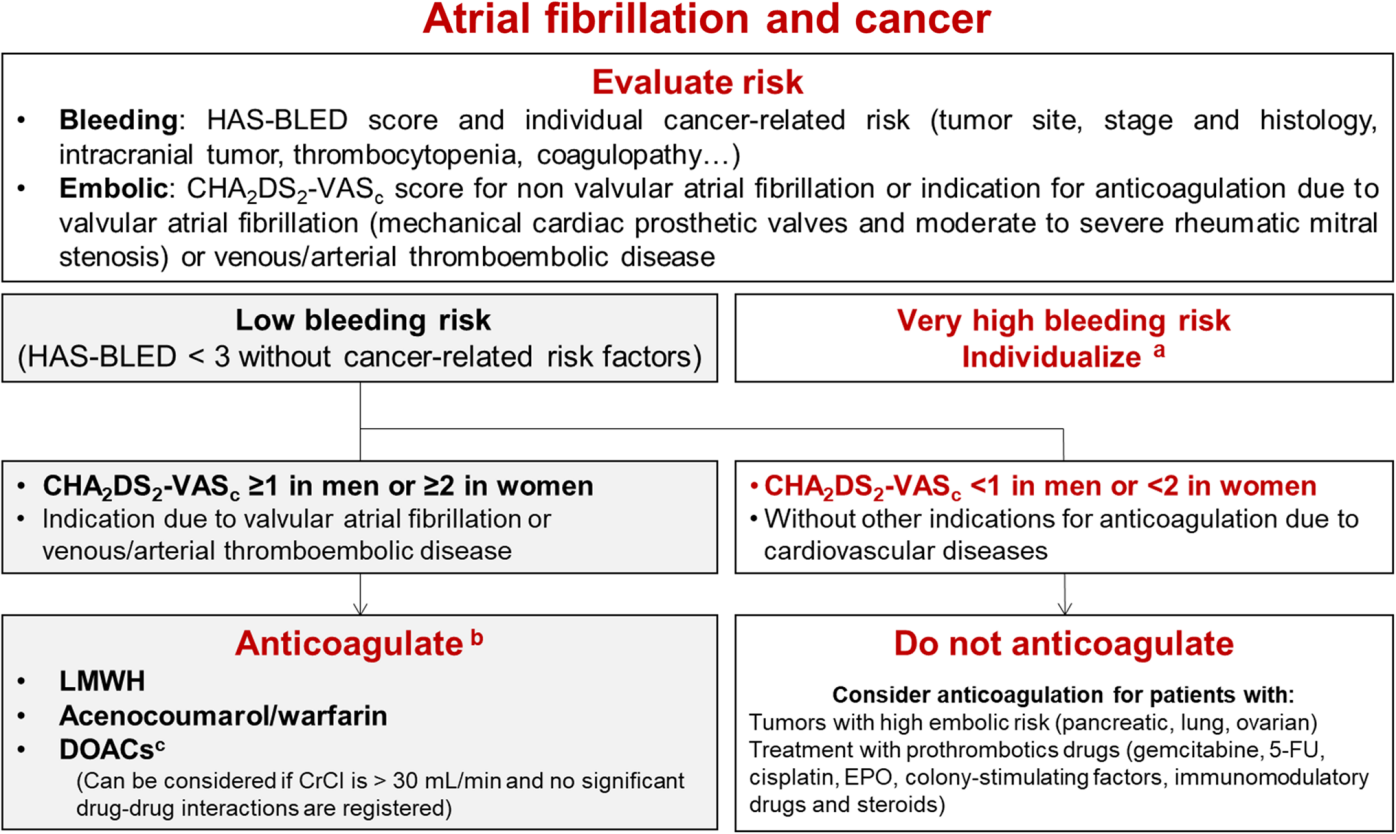
Algorithm for antithrombotic therapy in patients with cancer-related atrial fibrillation. Indication algorithm for anticoagulation in patients with cancer-related atrial fibrillation. Figure modified from [4]. *5-FU* 5-fluorouracil, *CHA2DS2-VASc* congestive heart failure, hypertension, age > 75 years (dual), diabetes mellitus, stroke (dual), vascular disease, age 65–74 years, and sex (female), *CrCl* creatinine clearance, *CYP* cytochrome P450, *DOACs* direct oral anticoagulants, *EPO* erythropoietin, *HAS-BLED* hypertension, abnormal renal and liver function, stroke, history of or predisposition to bleeding, labile international normalized ratio, age > 65 years, and concomitant use of drugs or alcohol, *LMWH* low-molecular-weight heparin, *P-gp* P-glycoprotein. ^a^For patients with very high bleeding risk and indication for anticoagulation the decision should be individualized. Considered in a multidisciplinary discussion if left atrial appendage occlusion. ^b^Anticoagulant selection depends on clinical status, comorbidities, and possible interactions with the patient’s anticancer therapy. ^c^Currently, there is limited scientific evidence on its use in patients under active anticancer therapy and atrial fibrillation

Atrial fibrillation (AF) is the most common sustained arrhythmia in cancer patients. Treating AF in patients receiving active anticancer therapy is a challenge owing to several factors, including the need for frequent procedures, malignancy-related risk factors for bleeding and/or thrombosis, drug–drug interactions, and the choice of anticoagulant treatment. Nowadays CHA_2_DS_2_VASc score is recommended to guide embolic risk stratification (Fig. 3) [4, 30–32]. Currently, there is limited scientific evidence on the use of direct oral anticoagulants in patients under active anticancer therapy; however, they can be considered as an alternative, in stable patients, if no significant drug–drug interaction was registered [32].

### Ischemic cardiomyopathy

**Fig. 4:**
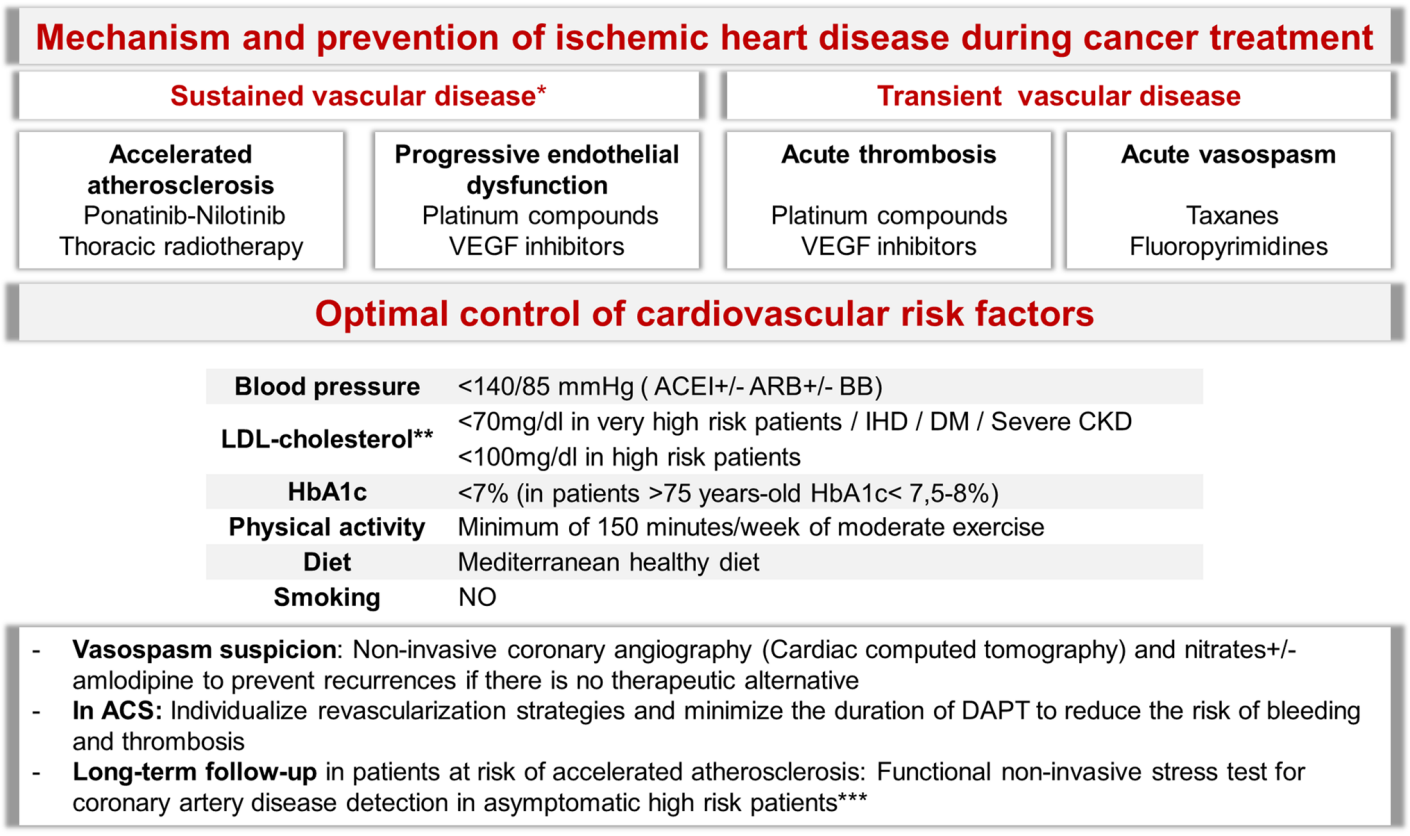
Mechanism and prevention of ischemic heart disease during cancer treatment. *Sustained vascular disease: more permanent and progressive disease, even after discontinuation of treatment. **Cardiovascular risk calculator: http://secardiologia.es/multimedia/apps/5696-calculadora-riesgo-cardiovascular. High-risk patients: radiotherapy in patients whose target volume includes at least part of the heart + 1 risk factor (< 15 or > 65 years-old at treatment; > 30 Gy or > 2 Gy/day; treatment with other cardiotoxic agents; previous ischemic heart disease, or preexisting classical cardiovascular risk factors). *DAPT* dual antiplatelet therapy, *ACS* acute coronary syndromes, *CT* computed tomography, *VEGF* vascular endothelial grow factor, *CVRF* cardiovascular risk factors, *ACEI* angiotensin converting enzyme inhibitors, *ARB* angiotensin II receptor antagonist, *BB* beta-blockers, *IHD* ischemic heart disease, *DM* diabetes mellitus, *CKD* chronic kidney disease

Despite the fact that cancer may induce ischemia by means of different mechanisms (Fig. 4), the most common ones are the sequelae from antitumor drugs and radiotherapy [3, 4, 33, 34].

Coronary heart disease (CHD) can debut as vasospasm, endothelial injury, or acute arterial thrombosis. Vasospasm has been reported during the administration of fluoropyrimidines or in the following days in up to 10% of the patients. Cisplatin induces endothelial dysfunction and arterial thrombosis, whereas VEGF pathway signaling inhibitors, such as bevacizumab, sunitinib, pazopanib, and sorafenib pose an increased risk of coronary thrombosis [36]. Radiotherapy entails a higher incidence of ischemic heart disease by means of endothelial injury, plaque rupture, and thrombosis [33, 37].

In individuals with pre-existing coronary disease who require treatment with 5-fluorouracil, etoposide, bleomycin, vinblastine, bevacizumab, sorafenib, and taxanes, it is mandatory that CV risk factors be controlled and development of symptoms suggestive of angina be meticulously assessed. In patients with coronary vasospasm, and normal or non-severe coronary artery disease, nitrates or calcium antagonists’ treatment minimizes vasospasm recurrence and avoids treatment interruptions. Triggers such as anemia should be minimized [3, 4].

### Other complications

Both prognosis and management of neoplastic pleural effusion depends on the underlying neoplasia. Pleural effusion has been reported during treatment with targeted drugs, such as imatinib. Therapeutic approach comprises diuretic´s administration; however refractory dose reduction treatment discontinuation may be necessary [38].

Myocarditis and pericarditis are rare complications of chemotherapy and/or radiotherapy. In both cases it can become complicated with pericardial effusion and needs non-steroidal anti-inflammatory drug treatment, combined with colchicine to reduce recurrences or pericardiocentesis if cardiac tamponade develops [3, 4, 37].

Pulmonary hypertension (PHT) is an uncommon, albeit serious CV side effect that appears after exposure to certain antineoplastic medications (TKIs, mainly dasatinib). Diagnosis is based on clinical evaluation, echocardiogram, and biomarkers (NT-proBNP). These patients require multidisciplinary evaluation to determine the best treatment strategy moving forward [3, 4, 39].

Radio-induced peripheral artery disease (PAD) mainly affects arteries and capillaries. Prevention depends on a strict CV risk factor control and treatment recommendations are similar other high-risk populations [40].

**Fig. 5:**
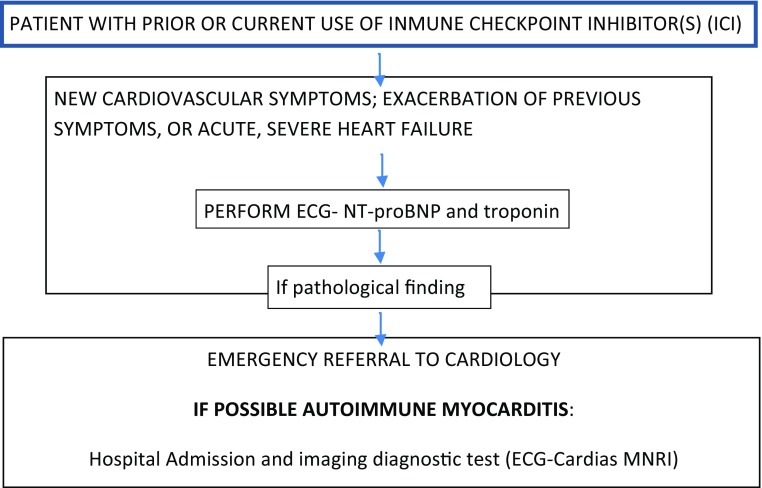
Management algorithm for suspected autoimmune myocarditis

Immune checkpoint inhibitors (ICI) are a new category of drugs that have had a great impact on the course of several advanced solid tumors. However, their use is related with immune system-mediated toxicities, including autoimmune myocarditis (AIM). Although cardiotoxic effects are uncommon, they are often associated with a high acute mortality risk. AIM prevalence increases under combination therapy and occurs more frequently during the first weeks of therapy [41]. It can manifest either as de novo HF or as an exacerbation of an already known HF. When AMI is suspected a prompt cardio-oncology consultation is required High-dose steroids are recommended in critical patients although there is currently little experience [42, 43]. Figure 5 summarizes clinical approach in suspected cases.

### Follow-up and treatment in long-term survivors

Cardiovascular disease and second cancers are the most common cause of mortality in cancer survivors [44]. Long-term survivors that have been treated with cardiotoxic treatments or radiotherapy should be informed of their increased risk for cardiovascular diseases (CVDs). Cardiovascular screening reduces the incidence of heart failure by 18% [45], but there is a lack of agreement about the optimal test for screening and frequency of testing. We propose the algorithm recommended by Carver et al. and the Spanish Working Group in Cardio-Oncology [4, 46].

During follow-up, education in long-term cancer survivors should be based on lifestyle modifications, to prevent and treat CV risk factors, and instructions to report early CV signs and symptoms (Table 1) [3, 4].

Patients who need treatment should be referred to the consultant cardiologist or to the cardio-oncology clinic. Early treatment with ACEIs, ARA-II, and/or BB improves HF prognosis [47].

#### Special situations

In patients with high risk for radiotherapy-induced cardiotoxicity, ECO every 5 years is recommended [4, 33].

Cardiac dysfunction may first become apparent during pregnancy. Women who want to become pregnant should be carefully evaluated before, during, and after pregnancy [4].

Patients with previous neck irradiation have an increased risk of stroke. Ultrasound scanning of carotid arteries to rule out the presence of subclinical atherosclerosis is recommended [3, 7].

Final recommendations, strength of recommendation and quality of evidence (Table 3).
